# Supplementary material for: Smartphone-Based Rapid Quantitative Detection Platform with Imprinted Polymer for Pb (II) Detection in Real Samples
Source: Polymers (Basel). 2024 May 28;16(11):1523. doi: 10.3390/polym16111523 (PMC11174601; doi:10.3390/polym16111523)
Supplement: Supplementary file 1 [file polymers-16-01523-s001.zip › polymers-3008414-supplementary.pdf]

# SUPPLEMENTARY MATERIAL

*Article*

# Smartphone-Based Rapid Quantitative Detection Platform with Imprinted Polymer for Pb (II) Detection in Real Samples

**Flor Meza Lopez[1], Christian Jacinto[6]; Jaime Vega-Chacón[1]; Juan C. Tuesta[5]; Gino Picasso[1]; Sabir Khan[2,4]; María D. P. T. Sotomayor[3,4,*]; Rosario López[2,*]**

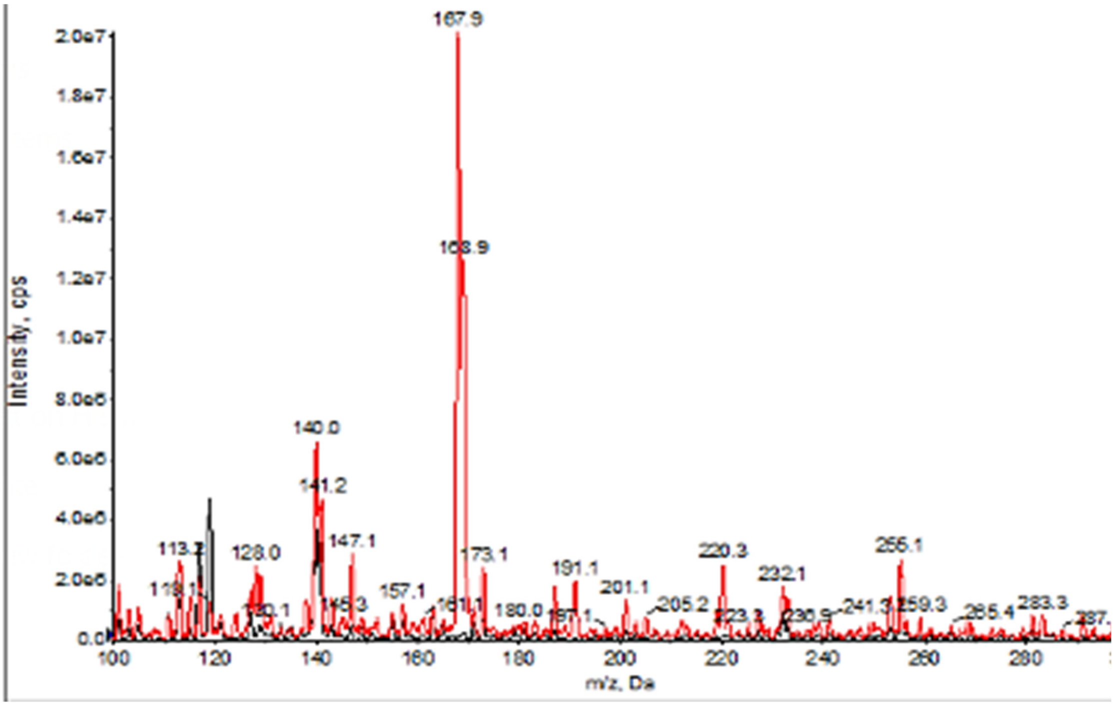


**Figure 1S.** Superposition of the spectra of the fresh rhodizonate solution (red) and the solution exposed to the environment (black).

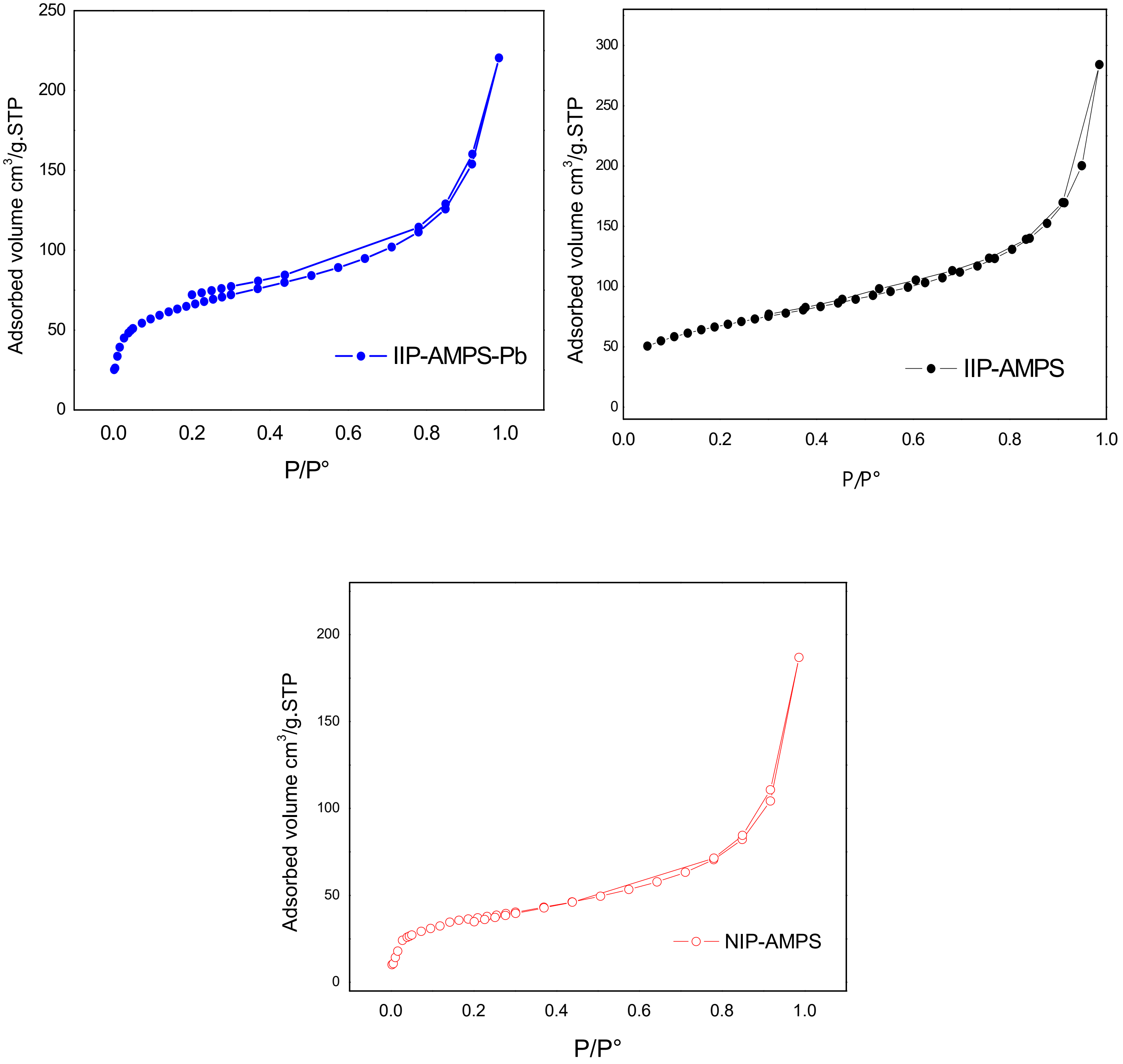


**Figure 2S.** $N_2$ adsorption isotherm of a) IIP-AMPS-Pb, b) IIP-AMPS and c) NIP-AMPS.

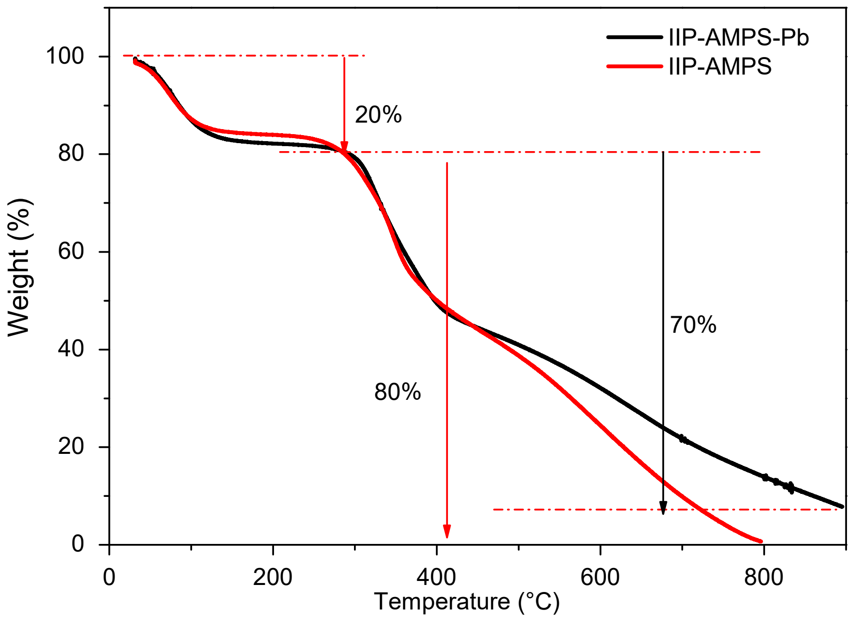


**Figure 3S**. TGA graphs of IIP-AMPS-Pb (black) and IIP-AMPS (red).

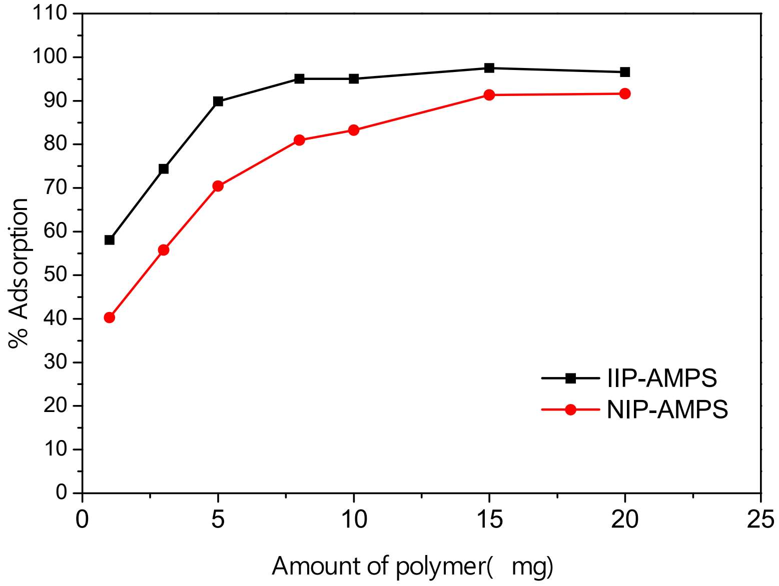


a)

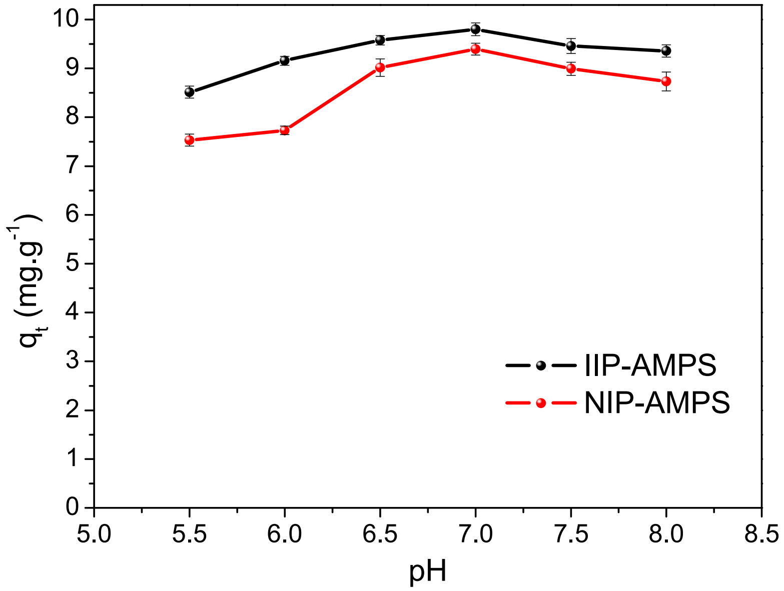


b)

**Figure 4S**. Effect of parameters on the adsorption of $Pb^{2+}$ (a) Effect of mass of polymer, (b) Effect of solution pH. Experimental conditions: initial concentration = 10 mg/L, T = 23 ± 1 °C

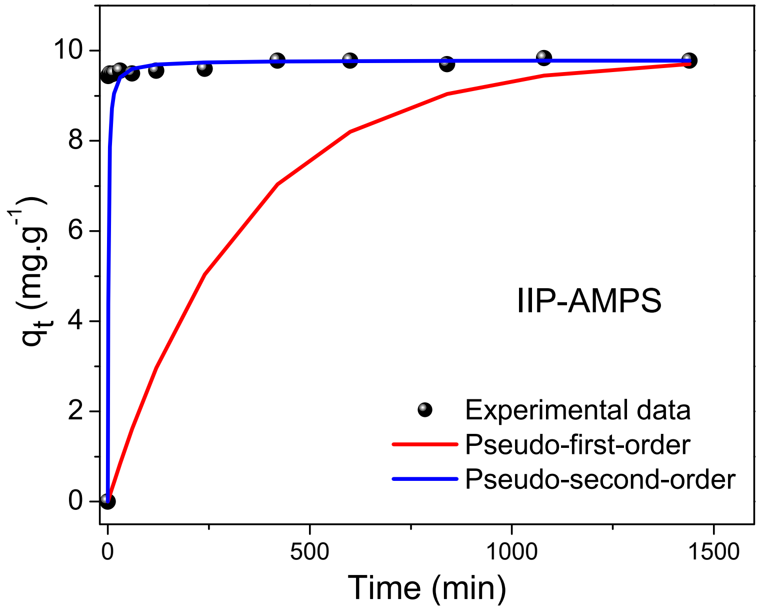


a)

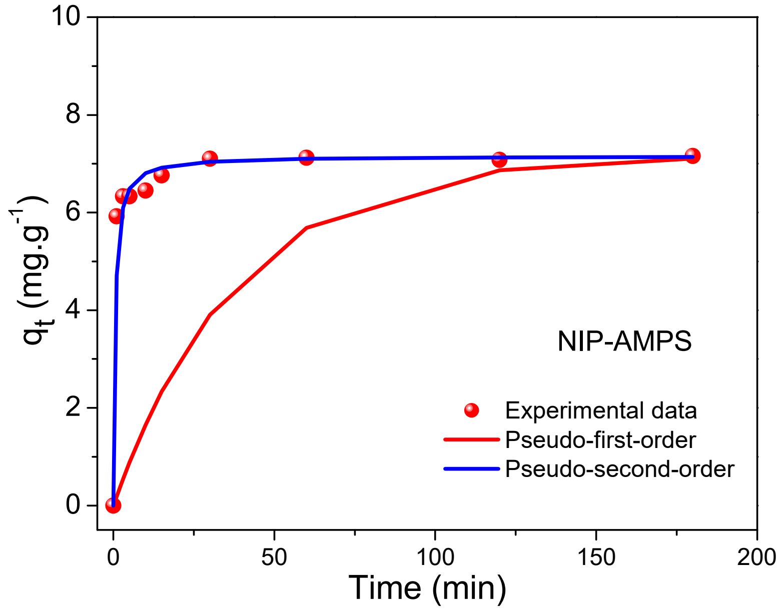


b)

**Figure 5S.** Adsorption kinetics for $Pb^{2+}$ with a nonlinear adjustment performed with the pseudofirst-order and pseudosecond-order model for (a) IIP-AMPS and (b) NIP-AMPSA. Experimental conditions: pH =6.0 ± 0.1, sorbent mass = 10 mg, initial concentration = 10 mg/L, T = 23 ± 1 °C

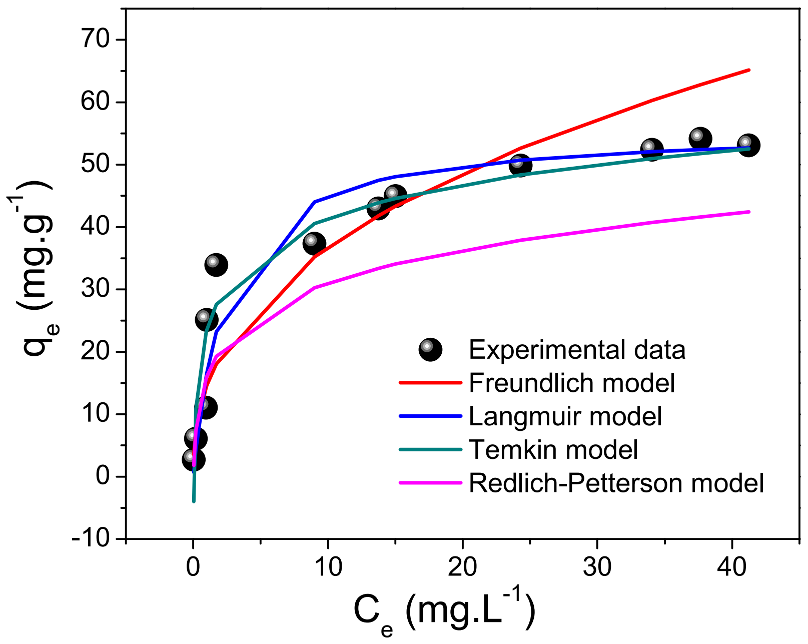


**Figure 6S**. Comparison of nonlinear modeling of the adsorption isotherm of IIP-AMPS.

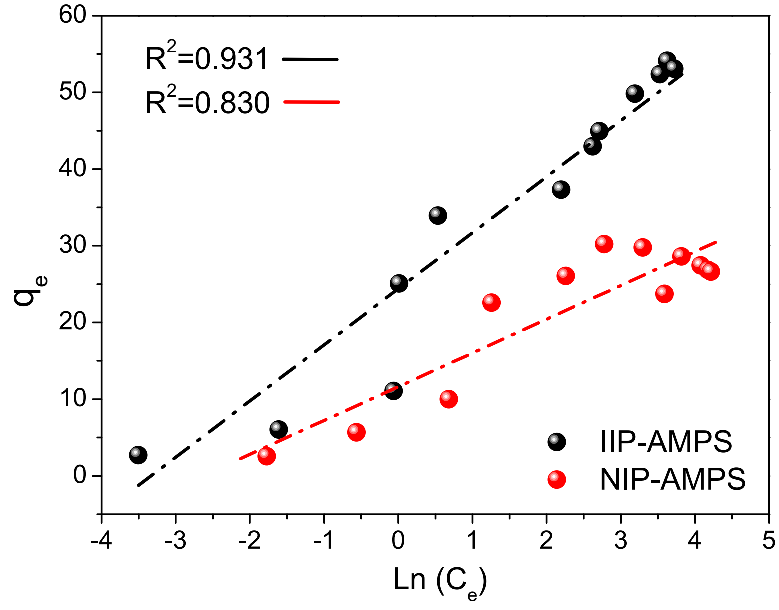


**Figure 7S**. Linear adjustment with Temkin model of the adsorption isotherm of IIP-AMPS and NIP-AMPS.

**Table 1S:** Textural parameters of the synthesized polymers obtained by the BET method.

| Material | $S_{BET}$ ($m^2\ g^{-1}$) | $S\mu p$ ($m^2\ g^{-1}$) | Smp ($m^2\ g^{-1}$) | Pore size (nm) | Pore volume ($cm^3 \cdot g^{-1}$) |
|---|---|---|---|---|---|
| **IIP-AMPS-Pb** | 169.23 | 20.42 | 148.25 | 10.95 | 0.298 |
| **IIP-AMPS** | 234.63 | 30.25 | 204.38 | 10.79 | 0.434 |
| **NIP-AMPS** | 144.99 | 22.45 | 122.54 | 10.99 | 0.209 |

**Table 2S.** Correlation parameters for the Pseudo-first-order kinetic models applied in the adsorption of Pb2+.

| | | Pseudo-first-order | | | |
|---|---|---|---|---|---|
| **Sorbents** | $q_{e\ (exp)}$ ($mg\ g^{-1}$) | $q_{e\ (cal)}$ ($mg\ g^{-1}$) | $K_2 \times 10^{-3}$ ($g\ mg^{-1}\ min^{-1}$) | $R^2$ | Error % |
| **IIP-AMPS** | 9.31 ± 0.12 | 0.40 | 2.17 | 0.460 | 2241.1 |
| **NIP-AMPS** | 7.16 ± 0.20 | 0.91 | 2.63 | 0.695 | 684.1 |

**Table 3S.** Freundlich, Langmuir, Elovich, Temkin, Dubinin-Radushkevich and Redlich-Peterson absorption isotherm constants for the adsorption of $Pb^{2+}$ on all polymeric materials.

| ISOTERMA DE FREUNDLICH | | | | | | | |
|---|---|---|---|---|---|---|---|
| **Isoterma** | **$R^2$** | **$\chi^2$** | **1/nF** | **log KF** | **$n_F$** | **$K_F$ mg.g$^{-1}$ (L.mg$^{-1}$)$^{1/n}$** | |
| IIP-AMPS | 0.909 | 27.55 | 0.40 | 1.16 | 2.48 | 14.53 | |
| NIP-AMPS | 0.834 | 24.89 | 0.36 | 0.88 | 2.75 | 7.66 | |
| ISOTERMA DE LANGMUIR | | | | | | | |
| **Isoterma** | **$R^2$** | **$\chi^2$** | **1/qm.KL** | **1/qm** | **qm (mg.g$^{-1}$)** | **KL (L.mg-1)** | |
| IIP-AMPS | 0.993 | 18.94 | 0.043 | 0.01795 | 55.72 | 0.418 | |
| NIP-AMPS | 0.993 | 6.25 | 0.044 | 0.03620 | 27.63 | 0.821 | |
| ISOTERMA DE ELOVICH | | | | | | | |
| **Isoterma** | **$R^2$** | **$\chi^2$** | **- 1/qm** | **ln($K_E q_m$)** | **qm (mg.g$^{-1}$)** | **$K_E$ (L.g$^{-1}$)** | |
| IIP-AMPS | 0.852 | 82.68 | -6.95E-02 | 4.23 | 14 | 4.76 | |
| NIP-AMPS | 0.638 | 192.78 | -1.07E-01 | 2.89 | 9 | 1.92 | |
| ISOTERMA DE TEMKIN | | | | | | | |
| **Isoterma** | **$R^2$** | **$\chi^2$** | **RT/$b_T$** | **(RT/$b_T$)ln$K_T$** | **$b_T$** | **$K_T$ (L.g$^{-1}$)** | |
| IIP-AMPS | 0.931 | -0.93 | 8 | 23 | 311.69 | 19.94 | |
| NIP-AMPS | 0.830 | 9.26 | 4 | 11 | 546.84 | 12.76 | |
| ISOTERMA DE DUBININ-RADUSHKEVICH | | | | | | | |
| **Isoterma** | **$R^2$** | **$\chi^2$** | **-$B_{DR}$** | **ln $q_s$** | **$B_{DR}$** | **$q_s$ (mg.g$^{-1}$)** | **E(J/mol)** |
| IIP-AMPS | 0.720 | 57.65 | -3.91E-08 | 3.57 | 3.91E-08 | 35.66 | 3577.85 |
| NIP-AMPS | 0.801 | 15.94 | -1.11E-07 | 3.15 | 1.11E-07 | 23.45 | 2121.55 |
| ISOTERMA DE REDLICH-PETERSON | | | | | | | |
| **Isoterma** | **$R^2$** | **$\chi^2$** | **$\alpha^\beta$/$K_{RP}$** | **$\beta$** | **$\alpha$ (L.μg$^{-1}$)$^\beta$** | **$K_{RP}$ (L.g$^{-1}$)** | **1/$K_{RP}$** |
| IIP-AMPS | 0.997 | 39.64 | 0.0379 | 0.800 | 3.58E+00 | 73.182 | 1.37E-02 |
| NIP-AMPS | 0.993 | 6.25 | 0.0362 | 1.000 | 8.21E-01 | 22.668 | 4.41E-02 |

**Table 4S.** Relative selectivity coefficient (K') of IIP-AMPS for $Pb^{2+}$.

| ION METAL | Ionic Radio (A°) | IIP | | NIP | | K' |
|---|---|---|---|---|---|---|
| | | $k_d$ | $k_{IIP}$ | $k_d$ | $k_{NIP}$ | |
| $Pb^{2+}$ | 1.19 | 21.78 | | 2.57 | | |
| $Ni^{2+}$ | 0.73 | 0.24 | 40.92 | 0.58 | 4.84 | 8.46 |
| $Cu^{2+}$ | 0.69 | 0.16 | 321.05 | 0.67 | 4.14 | 77.57 |
| $Cd^{2+}$ | 0.97 | 0.29 | 72.70 | 1.86 | 1.31 | 55.39 |
| $Zn^{2+}$ | 0.74 | 0.17 | 163.47 | 1.19 | 2.29 | 71.48 |
| $Fe^{3+}$ | 0.64 | 0.17 | 67.24 | 0.62 | 3.52 | 19.09 |
| $Ca^{2+}$ | 0.99 | 0.08 | 125.24 | 0.84 | 2.95 | 42.50 |
| $Hg^{2+}$ | 1.10 | 0.14 | 155.57 | 0.89 | 2.89 | 53.87 |
